# Diagnostic performance and generalizability of a clinical-ultrasound radiomics model for predicting extrathyroidal extension in thyroid carcinoma: a retrospective study

**DOI:** 10.1080/07853890.2026.2650862

**Published:** 2026-03-30

**Authors:** Tengfei Zheng, Zhen Xu, Litao Hu, Yan Zhang, Xiaofen Liu, Xinjia Jiang, Yutian Liao, Pan Xu, Xinchun Yuan

**Affiliations:** a Department of Ultrasound, The First Affiliated Hospital, Jiangxi Medical College, Nanchang University, Nanchang, Jiangxi, China; b Department of Ultrasound, Huashan Hospital, Fudan University, Shanghai, China

**Keywords:** Thyroid carcinoma, extrathyroidal extension, ultrasound, radiomics

## Abstract

**Background:**

Ultrasound plays a crucial role in the preoperative evaluation of thyroid carcinoma and its extrathyroidal extension (ETE). This study aims to develop and validate a model integrating ultrasound radiomics and clinical factors for predicting ETE.

**Methods:**

In this retrospective study, data from 420 patients who underwent thyroid cancer surgery at the First Affiliated Hospital of Nanchang University were reviewed. Patients were categorized into non-ETE and ETE groups based on postoperative pathology and randomly divided (7:3 ratio) into training and internal validation cohorts. An external test cohort included 70 patients from Huashan Hospital, Fudan University. Radiomics features were extracted from preoperative ultrasound images of the entire tumor region. Feature selection was performed using Least Absolute Shrinkage and Selection Operator (LASSO) logistic regression to construct a radiomics signature. A nomogram was subsequently developed by combining the radiomics score with clinical-ultrasound factors. The model’s predictive performance was evaluated using receiver operating characteristic (ROC) curve analysis and decision curve analysis (DCA) across all cohorts.

**Results:**

The combined model demonstrated excellent calibration and discrimination, with areas under the curve (AUC) of 0.898, 0.873, and 0.857 in the training, internal validation, and external test cohorts, respectively. DCA confirmed the superior clinical utility of the radiomics nomogram compared to models using clinical-ultrasound factors alone. Subgroup analysis further revealed the model’s robust performance across different tumor sizes, ETE degrees, and Hashimoto’s thyroiditis status.

**Conclusion:**

The ultrasound radiomics-based nomogram shows favorable performance and high accuracy in predicting ETE, suggesting its potential as a valuable preoperative auxiliary diagnostic tool.

## Introduction

Thyroid cancer ranks as the seventh most common cancer globally, with its incidence having increased markedly over the past four decades [1]. Papillary thyroid carcinoma (PTC) is the most prevalent histological type, accounting for approximately 85% of cases, and is generally associated with a favorable prognosis [2,3]. However, the prognosis of PTC patients is significantly compromised by the presence of cervical lymph node metastasis, distant metastasis, or extrathyroidal extension (ETE). Consequently, ETE is incorporated as a key prognostic indicator in several risk stratification systems for differentiated thyroid carcinoma (DTC), such as the GAMES (Gender, Age, Metastasis, Extrathyroidal extension, Size) [4] and MACIS (Metastasis, Age, Completeness of resection, Invasion, Size) systems [5]. Notably, the 15-year survival rate for PTC patients with ETE is significantly lower than that for patients without ETE [6].

Currently, histopathological examination following biopsy or surgery remains the gold standard for diagnosing ETE [7]. Various imaging modalities, including magnetic resonance imaging (MRI), computed tomography (CT), single-photon emission computed tomography (SPECT), and ultrasonography (US), are employed for the preoperative assessment of ETE. Among these, US has emerged as the most frequently used imaging technique for PTC patients due to its cost-effectiveness, non-invasive nature, and the absence of ionizing radiation. Therefore, this study focuses on US-based radiomics analysis to predict ETE. Previous studies [8–11] have identified corresponding ultrasonographic features for involvement of the strap muscles, trachea, and recurrent laryngeal nerve (RLN) in ETE, providing a crucial reference for its preoperative diagnosis. However, the specificity of US-based ETE diagnosis remains limited (40%-50%), despite its relatively high sensitivity (60%-70%) [12].

Radiomics involves the high-throughput extraction of sub-visual features from medical images for quantitative disease analysis [13–16]. It has been increasingly applied in oncology research for tasks including tumor diagnosis, prognosis prediction, and genomic analysis. To identify the most informative radiomics features and construct a predictive model, we employed Least Absolute Shrinkage and Selection Operator (LASSO) regression, a machine learning method that selects features by shrinking the coefficients of less important predictors to zero, thereby reducing overfitting and enhancing model generalizability. The radiomics score (Rad-score), calculated as a linear combination of the selected features weighted by their respective LASSO coefficients, serves as a quantitative imaging biomarker that reflects an individual patient’s radiomics signature—higher Rad-scores indicate a greater likelihood of ETE. Therefore, this study aims to develop a combined model based on US radiomics to predict ETE in PTC, thereby addressing the limitations of conventional US evaluation and potentially assisting clinicians in selecting the most appropriate treatment strategies for PTC patients.

## Materials and methods

### Study population and design

This retrospective study was approved by the Institutional Review Board (approval number: IIT [2024]-724) and was conducted in accordance with the principles of the Declaration of Helsinki of 1964 and its later amendments. The requirement for informed consent was waived due to the retrospective nature of the study.

We retrospectively enrolled patients with PTC who were treated at The First Affiliated Hospital of Nanchang University (Institution 1) between January 2022 and December 2024. An external validation cohort consisted of patients from Huashan Hospital, Fudan University (Institution 2) between November 2023 and September 2024.

The inclusion criteria were as follows: (1) patients who underwent total or partial thyroidectomy for thyroid cancer; (2) patients who underwent preoperative thyroid ultrasonography (US) evaluation.

The exclusion criteria were: (1) patients undergoing surgery for recurrent thyroid cancer; (2) patients with incomplete surgical or pathological reports; (3) patients with poor-quality US images, specifically, this included cases with: image blurring or low resolution that hindered the identification of lesion boundaries; significant artifacts (such as acoustic shadowing, reverberation, or gas interference) obscuring key areas of the lesion; or insufficient grayscale contrast, making it difficult to differentiate the lesion from surrounding tissues. (4) patients with evidence of distant metastasis; (5) patients with incomplete preoperative serological test results; and (6) patients with incomplete clinical data or missing follow-up records.

**Figure 1.**
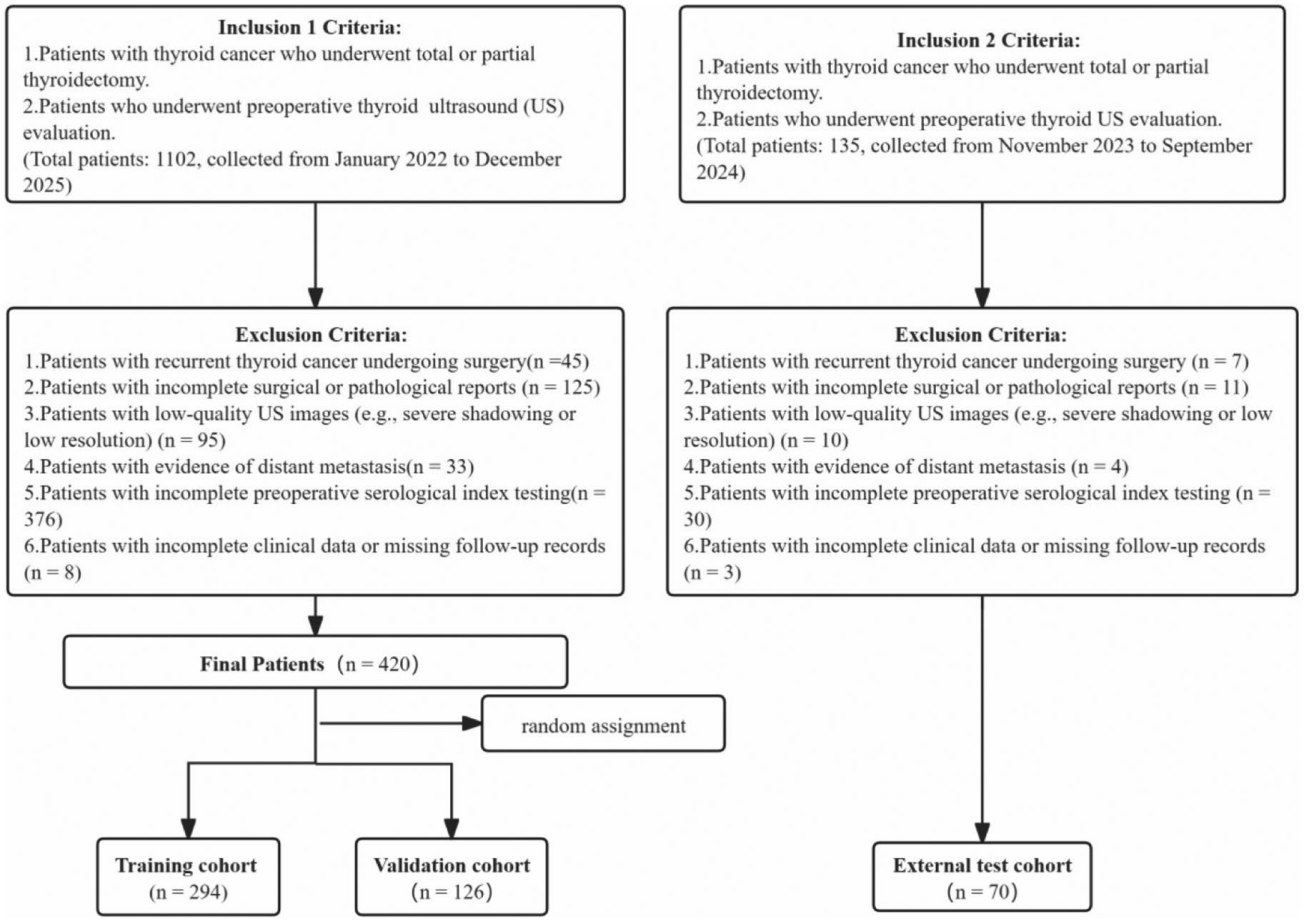
Patient Inclusion and Exclusion Flowchart Criteria. Flowchart showing criteria for including/excluding thyroid cancer patients, and splitting into training, validation, external test cohorts. ETE: extrathyroidal extension.

Enrolled patients were randomly allocated into a training set (*n* = 294) and an internal test set (*n* = 126) at a ratio of 7:3. An independent external validation cohort consisting of 70 patients was also established. The study flowchart is presented in Figure 1.

The following baseline data and clinical indicators—all obtained preoperatively—were collected: demographic characteristics (age, sex); tumor clinical features (tumor size, primary location); and thyroid function with related antibody levels. Age and tumor diameter were dichotomized using thresholds of ≥55 years and ≥2 cm, respectively. These thresholds were determined with reference to the pivotal criteria for tumor staging and risk stratification outlined in the American Joint Committee on Cancer (8th edition) Thyroid Cancer TNM Staging System and the American Thyroid Association Management Guidelines for Differentiated Thyroid Cancer. Additionally, cervical lymph node metastasis status, as confirmed by postoperative pathology, was included in the analysis.

The specific thyroid function and antibody assays, along with their reference ranges [17,18], were as follows: free triiodothyronine (fT3, reference range: 2.50–3.90 pg/mL), free thyroxine (fT4, reference range: 0.60–1.60 ng/dL), thyroid-stimulating hormone (TSH, reference range: 0.30–5.60 μIU/mL), anti-thyroglobulin antibody (Tg-Ab, reference range: 0.0–60.0 IU/mL), anti-thyroid peroxidase antibody (TPO-Ab, reference range: 0.0–60.0 IU/mL), and serum thyroglobulin (Tg, reference range: 1.40–78 ng/mL).

The extent and degree of tumor invasion were comprehensively and accurately assessed through a combination of serial pathological sectioning techniques, postoperative histopathological examination findings, and intraoperative gross observations.According to the 8th edition of the American Joint Committee on Cancer (AJCC) staging manual [3], tumors with invasion of the perithyroidal soft tissues, including microscopic muscular invasion, were diagnosed as minimal extrathyroidal extension (mETE). Tumors with gross invasion into the strap muscles or major neck structures (subcutaneous soft tissues, larynx, trachea, esophagus, recurrent laryngeal nerve, prevertebral fascia, or carotid artery/mediastinal vessels) were diagnosed as gross extrathyroidal extension (gETE). For the purpose of this study, both mETE and gETE were categorized collectively as ETE for subsequent analysis.

### Instrumentation and examination protocol

Ultrasonography was performed using the following systems: Philips EPIQ 7 (Philips Healthcare), Toshiba Aplio 500 (Canon Medical Systems), Mindray Resona 7, Mindray DC-8 (Shenzhen Mindray Bio-Medical Electronics Co., Ltd.), Siemens ACUSON Sequoia, and Supersonic Aixplorer. Patients were placed in the supine position with the neck fully exposed for the preoperative US examination. All US images were acquired by physicians with over five years of experience in thyroid ultrasound diagnosis.

In this study, ultrasound features refer to the specific imaging characteristics assessed by experienced radiologists on preoperative US images, including both qualitative and quantitative parameters. The acquired US images were used to document the following characteristics: the maximum diameter of the tumor, multifocality, bilaterality, location, nodule contact with the thyroid capsule, tumor-tracheal angle, contact with the tracheoesophageal groove, and the presence of any sonographically suspicious lymph nodes. Findings were also assessed and recorded according to the China Thyroid Imaging Reporting and Data System (C-TIRADS).

### Radiomics analysis

#### Image preprocessing

Prior to radiomics feature extraction, standardized preprocessing was performed on all two-dimensional ultrasound images. The steps were as follows: ① Images were resampled to a consistent pixel spacing of 1.0 mm × 1.0 mm using linear interpolation to eliminate resolution variability. ② Gray-level intensities within each region of interest (ROI) were discretized using a fixed-bin number method with 64 bins to reduce sensitivity to noise. ③ A mild two-dimensional Gaussian filter (σ = 0.5 mm) was applied to each ROI to suppress inherent speckle noise. ④ Z-score normalization (mean = 0, standard deviation = 1) was applied to the pixel intensities within each individual ROI to minimize variability arising from differences in scan gain settings.

#### Feature extraction

One experienced radiologist with over 5 years of expertise in thyroid ultrasound diagnosis manually delineated the region of interest (ROI) on the largest and clearest two-dimensional (2D) cross-sectional image of the tumor using 3D-Slicer software (version 5.9.0). The ROI was drawn closely along the tumor boundary to encompass the entire solid tumor area while excluding cystic changes, coarse calcifications, and surrounding normal thyroid tissue. Radiomics features are defined as high-dimensional, quantitative imaging biomarkers extracted through computer algorithms, which capture tissue heterogeneity and textural information imperceptible to the human eye. In this study, radiomics features were subsequently extracted using the radiomics module integrated within the 3D-Slicer platform. A total of 849 radiomics features were extracted from each ROI. These features comprised six categories: first-order statistics, Gray Level Dependence Matrix (GLDM), Gray Level Co-occurrence Matrix (GLCM), Gray Level Run Length Matrix (GLRLM), Gray Level Size Zone Matrix (GLSZM), and Neighboring Gray Tone Difference Matrix (NGTDM). Features were derived from both the original images and from images transformed using wavelet filters.

Inter- and intra-observer reproducibility of feature extraction was assessed using the intraclass correlation coefficient (ICC). To calculate the ICC, a random subset of US images from 50 patients was re-segmented two weeks later by the same radiologist (Reader 1) and by a second independent radiologist (Reader 2). The primary segmentation for all images used in the final analysis was performed exclusively by Reader 1.

#### Feature selection

To avoid optimistic bias, all feature selection procedures were strictly confined to the training set (*n* = 294) and conducted independently. Neither the internal validation set nor the external test set was involved in any step of feature selection or parameter tuning.

The feature selection process was conducted as follows. First, based on the training set data, the intraclass correlation coefficient (ICC) was utilized to assess the reproducibility of all initially extracted radiomics features. Only features demonstrating high reproducibility (ICC ≥ 0.80) were retained for subsequent analysis. Subsequently, a Pearson correlation coefficient matrix was computed for the retained features within the training cohort. Feature pairs exhibiting a high degree of collinearity (absolute correlation coefficient |r| > 0.7) were identified, and one feature from each such pair was randomly removed to ensure feature independence for the subsequent modeling. Finally, on the training set, the Least Absolute Shrinkage and Selection Operator (LASSO) regression algorithm was applied for further feature selection. The optimal penalty parameter was tuned *via* ten-fold cross-validation based on the minimum binomial deviance. Features with non-zero coefficients were selected, and these coefficients were defined as the weights representing the relevance of each feature to ETE. The radiomics score (Rad-score) for each patient was then computed as a linear combination of the selected features weighted by their respective LASSO coefficients. This Rad-score was used to build the final radiomics model.

#### Model development and performance evaluation

Three predictive models were developed: a clinical model, a radiomics model, and a combined model. In the training set, variables demonstrating statistical significance in univariate logistic regression analysis were subsequently included in a multivariate analysis to construct both the clinical model and the combined model. The Delong test was employed to compare the area under the curve (AUC) among the three models. Calibration curves were employed to evaluate the calibration performance of the nomogram, while the Brier score was used to quantitatively assess the model’s overall predictive accuracy. The finalized model was subsequently applied to the internal validation and external test sets for independent performance evaluation. The discriminative ability and clinical utility of the model were further analyzed using receiver operating characteristic (ROC) curve analysis and decision curve analysis (DCA).

### Statistical analysis

All statistical analyses were performed using R software (version 4.4.2; R Foundation for Statistical Computing). Continuous variables following a normal distribution are presented as mean ± standard deviation and were compared using independent one-way ANOVA. Non-normally distributed continuous variables are expressed as median (interquartile range) and were compared using the Kruskal-Wallis test. Categorical data are summarized as frequency (percentage), and group comparisons were conducted using the Chi-square test or Fisher’s exact test, as appropriate.

Multivariable logistic regression analysis was employed to identify independent predictors among the clinical variables, with a *p*-value < 0.05 considered statistically significant. Differences in the area under the receiver operating characteristic curve (AUC) between subgroups were analyzed using the Hanley-McNeil Z-test, with a two-sided *p*-value < 0.05 defined as statistically significant.

## Results

### Clinical characteristics

The training and internal validation cohort (Institution 1) comprised 420 PTC patients, aged 12–75 years (mean age 45.22 ± 11.30 years), including 83 males and 337 females. Among them, 263 patients had ETE (199 with minimal ETE and 64 with gross ETE), while 157 showed no ETE. The training cohort included 294 patients (57 males, 237 females), with 187 presenting ETE and 107 without ETE, and a mean age of 45.20 ± 11.50 years. The internal validation cohort consisted of 126 patients (26 males, 100 females; mean age 45.25 ± 10.86 years), including 76 with ETE and 50 without ETE. The external test cohort (Institution 2) included 70 patients aged 17–74 years (mean age 43.04 ± 13.78 years), comprising 19 males and 51 females. Of these, 39 had ETE (28 minimal ETE, 11 gross ETE) and 31 showed no ETE.

The baseline characteristics of the patients are summarized in Table 1. With the exception of free thyroxine (fT4) levels (*p* = 0.024), no statistically significant differences were observed in baseline characteristics among the three cohorts (all *p* > 0.05), indicating generally balanced and comparable baseline data across groups. Although a minor distributional difference was noted in fT4 levels, its magnitude was small, suggesting limited potential impact on subsequent model validation and result stability.

**Table 1.** Baseline patient characteristics in the training, validation, and external validation sets.

	Training set	Internal test set	External test set	*p*
Patient	294	126	70	–
Gender (%)				0.356
Male	57 (19.39%)	26 (20.63%)	19 (27.14%)	
Female	237 (80.61%)	100 (79.37%)	51 (72.86%)	
Age, mean ± SD	45.2 ± 11.5	45.25 ± 10.86	43.04 ± 13.78	0.354
< 55 years	230 (78.23%)	98 (77.78%)	54 (77.14%)	
≥ 55 years	64 (21.77%)	28 (22.22%)	16 (22.86%)	
Longest diameter ≥ 2 cm (%)				0.233
No	243 (82.65%)	96 (76.19%)	59 (84.29%)	
Yes	51 (17.35%)	30 (23.81%)	11 (15.71%)	
Multifocality (%)				0.826
No	151 (51.36%)	67 (53.17%)	34 (48.57%)	
Yes	143 (48.64%)	59 (46.83%)	36 (51.43%)	
Bilaterality (%)				0.987
No	199 (67.69%)	86 (68.25%)	48 (68.57%)	
Yes	95 (32.31%)	40 (31.75%)	22 (31.43%)	
Isthmus (%)				0.573
No	263 (89.46%)	114 (90.48%)	60 (85.71%)	
Yes	31 (10.54%)	12 (9.52%)	10 (14.29%)	
Central lymph node metastasis (%)				0.721
No	139 (47.28%)	65 (51.59%)	34 (48.57%)	
Yes	155 (52.72%)	61 (48.41%)	36 (51.43%)	
Lateral lymph node metastasis (%)				0.675
No	239 (81.29%)	98 (77.78%)	55 (78.57%)	
Yes	55 (18.71%)	28 (22.22%)	15 (21.43%)	
Hashimoto’s thyroiditis (%)				0.595
No	150 (51.02%)	71 (56.35%)	36 (51.43%)	
Yes	144 (48.98%)	55 (43.65%)	34 (48.57%)	
Contact of the nodule with the thyroid capsule (%)				0.225
No Contact	57 (19.39%)	29 (23.02%)	18 (25.71%)	
Capsular contact	132 (44.9%)	60 (47.62%)	34 (48.57%)	
Contour bulging	86 (29.25%)	24 (19.05%)	14 (20%)	
Replacement of strap muscle	19 (6.46%)	13 (10.32%)	4 (5.71%)	
Angle between Tumor and Trachea (%)				0.308
No contact	159 (54.08%)	76 (60.32%)	39 (55.71%)	
Acute angle	63 (21.43%)	14 (11.11%)	14 (20%)	
Right angle	18 (6.12%)	7 (5.56%)	5 (7.14%)	
Obtuse angle	54 (18.37%)	29 (23.02%)	12 (17.14%)	
Contact of the nodule with the TEG (%)				0.630
No contact	156 (53.06%)	75 (59.52%)	39 (55.71%)	
Abutting TEG	75 (25.51%)	28 (22.22%)	20 (28.57%)	
Protrusion into TEG	63 (21.43%)	23 (18.25%)	11 (15.71%)	
C-TIRADS 3 (%)				0.273
No	277 (94.22%)	121 (96.03%)	69 (98.57%)	
Yes	17 (5.78%)	5 (3.97%)	1 (1.43%)	
C-TIRADS 4 A (%)				0.604
No	185 (62.93%)	85 (67.46%)	47 (67.14%)	
Yes	109 (37.07%)	41 (32.54%)	23 (32.86%)	
C-TIRADS 4B (%)				0.971
No	181 (61.56%)	77 (61.11%)	42 (60%)	
Yes	113 (38.44%)	49 (38.89%)	28 (40%)	
C-TIRADS 4 C (%)				0.125
No	264 (89.8%)	108 (85.71%)	57 (81.43%)	
Yes	30 (10.2%)	18 (14.29%)	13 (18.57%)	
C-TIRADS 5 (%)				0.728
No	269 (91.5%)	113 (89.68%)	65 (92.86%)	
Yes	25 (8.5%)	13 (10.32%)	5 (7.14%)	
fT3 (pg/mL)	3.14 (2.92,3.41)	3.17 (2.87,3.41)	3.18 (2.9,3.54)	0.608
fT4 (ng/dL)	1.24 (1.12,1.37)	1.29 (1.18,1.42)	1.29 (1.18,1.37)	0.027
TSH (μIU/mL)	1.77 (1.18,2.63)	1.76 (1.18,2.64)	1.81 (1.2,2.85)	0.653
TG-Ab (IU/mL)	18.1 (14.4,69.65)	17.5 (14.43,34.12)	17.88 (13.41,74.56)	0.723
TPO-Ab (IU/mL)	24.2 (16.08,36.41)	25.4 (15.25,35.48)	24.24 (18.13,45.87)	0.719
Tg (ng/mL)	16.8 (6.39,44.53)	15 (5.02,33.73)	13.72 (3.87,28.06)	0.329

Abbreviations: TEG (tracheoesophageal groove); C-TIRADS (Chinese – Thyroid Imaging Reporting and Data System); fT3 (free triiodothyronine); fT4 (free thyroxine); TSH (thyroid – stimulating hormone); TG – Ab (thyroglobulin antibody); TPO – Ab (thyroid peroxidase antibody); Tg (thyroglobulin).

Univariate logistic regression analysis identified the following factors as significantly associated with ETE (*p* < 0.05): age ≥55 years, central lymph node metastasis, lateral lymph node metastasis, bilateral tumor, tumor multifocality, larger tumor diameter (≥2 cm), isthmic tumor, nodule contact with the thyroid capsule, tumor-tracheal angle, contact with the tracheoesophageal groove, and C-TIRADS categories 4a, 4c, and 5. Detailed results are provided in Table 2.

**Table 2.** Univariable and multivariable logistic regression analysis.

	Univariable analysis		Multivariable analysis((clinical model parameters)		Multivariable analysis(combined model parameters))	
Variable	OR(95%CI)	*p*	OR(95%CI)	*p*	OR(95%CI)	*p*
≥ 55 years	2.178(1.177–4.230)	0.016	2.901(1.292–6.797)	0.012	3.867(1.662–9.548)	0.002
Longest diameter ≥ 2 cm	11.971(4.237–50.200)	< 0.001	–	–	–	–
Bilaterality	2.474(1.441–4.375)	0.001	–	–	–	–
Isthmus	6.105(2.094–25.980)	0.004	–	–	–	–
Central lymph	2.514(1.549–4.119)	< 0.001	–	–	–	–
Lateral lymph	5.977(2.650–16.050)	< 0.001	3.301(1.131–10.700)	0.035	2.607(0.904–8.445)	0.089
Contact of with the TEG	6.709(4.119–11.770)	< 0.001	2.545(1.171–5.916)	0.022	2.642(1.234–6.083)	0.016
Angle	4.750(3.061–8.080)	< 0.001	1.900(1.047–3.751)	0.046	1.737(0.968–3.385)	0.080
Contact with the thyroid capsule	2.743(1.962–3.934)	< 0.001	1.787(1.172–2.776)	0.008	1.677(1.056–2.709)	0.031
C-TIRADS 4 A	0.360(0.219–0.589)	< 0.001	–	–	–	–
C-TIRADS 4 C	5.850(2.001–24.930)	0.004	4.601(1.245–22.860)	0.035	3.207(0.841–16.210)	0.113
C-TIRADS 5	15.607(3.224–281.000)	0.008	–	–	–	–
TSH (μIU/mL)	1.245(1.027–1.548)	0.040	–	–	–	–
Rad_Score	3.955(2.730–6.044)	< 0.001	–	–	2.894(1.890–4.727)	< 0.001

Abbreviations: OR (odds ratios); CI (confidence interval).

### Feature selection and radiomics signature model construction

**Figure 2.**
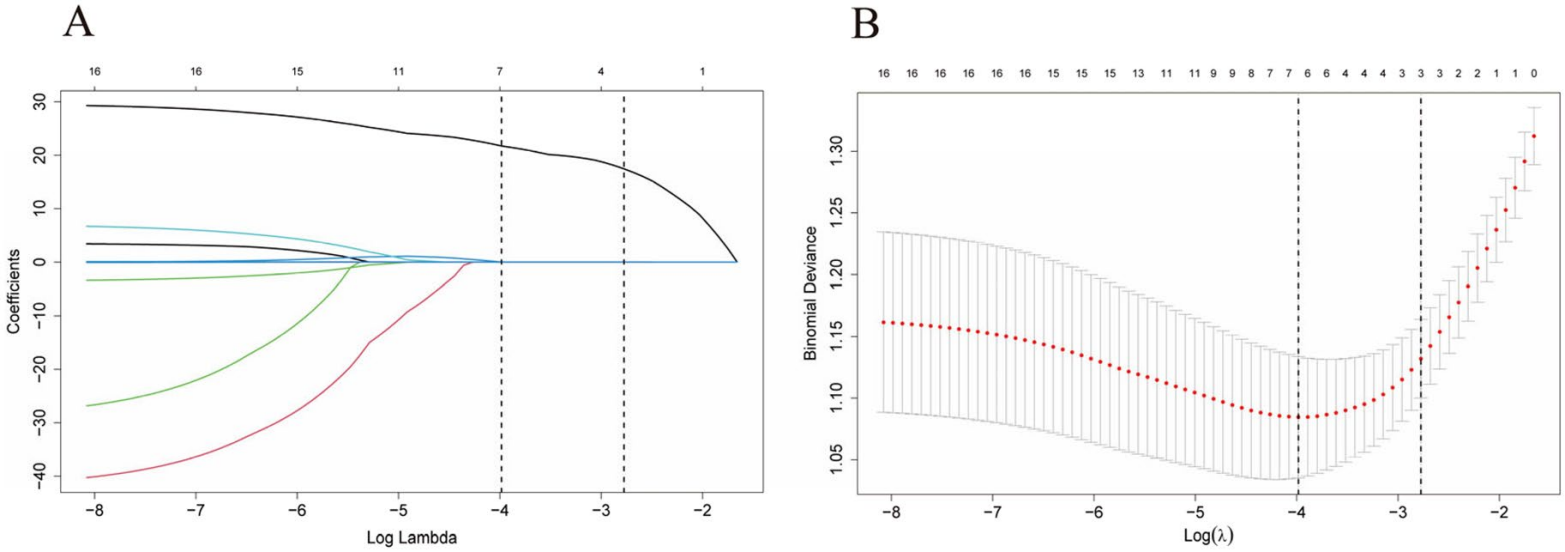
Radiomic signature selection using the Least Absolute Shrinkage and Selection Operator (LASSO) regression model. (a) Trajectory plot of the coefficients for the 16 candidate radiomic features against the log(λ) values. A ten-fold cross-validation was employed to determine the optimal λ value. The vertical dashed line is drawn at the optimal log(λ) value of 0.022, which resulted in a radiomic signature comprising 7 features with non-zero coefficients. The top x-axis indicates the number of features retained in the model at different λ values. (b) The partial likelihood deviance for the LASSO model across log(λ) values. The vertical dashed line corresponds to the optimal λ value selected *via* ten-fold cross-validation in (A). The top x-axis denotes the number of non-zero coefficients in the model at corresponding λ values.

From the preprocessed images, a total of 849 preliminary radiomics features were extracted using the PyRadiomics module within 3D-Slicer. First, 669 features with low reproducibility (ICC < 0.80) were excluded based on intra- and inter-observer consistency assessment, retaining 180 stable features. Subsequent elimination of redundant features based on a Pearson correlation coefficient threshold (|r| ≥ 0.7) yielded 16 key features. Finally, the least absolute shrinkage and selection operator (LASSO) regression was applied, identifying 7 features with non-zero coefficients (Figure 2). The radiomics signature model was then constructed through a linear combination of these selected features weighted by their corresponding coefficients.

### Performance comparison of radiomics, clinical, and combined models

**Figure 3.**
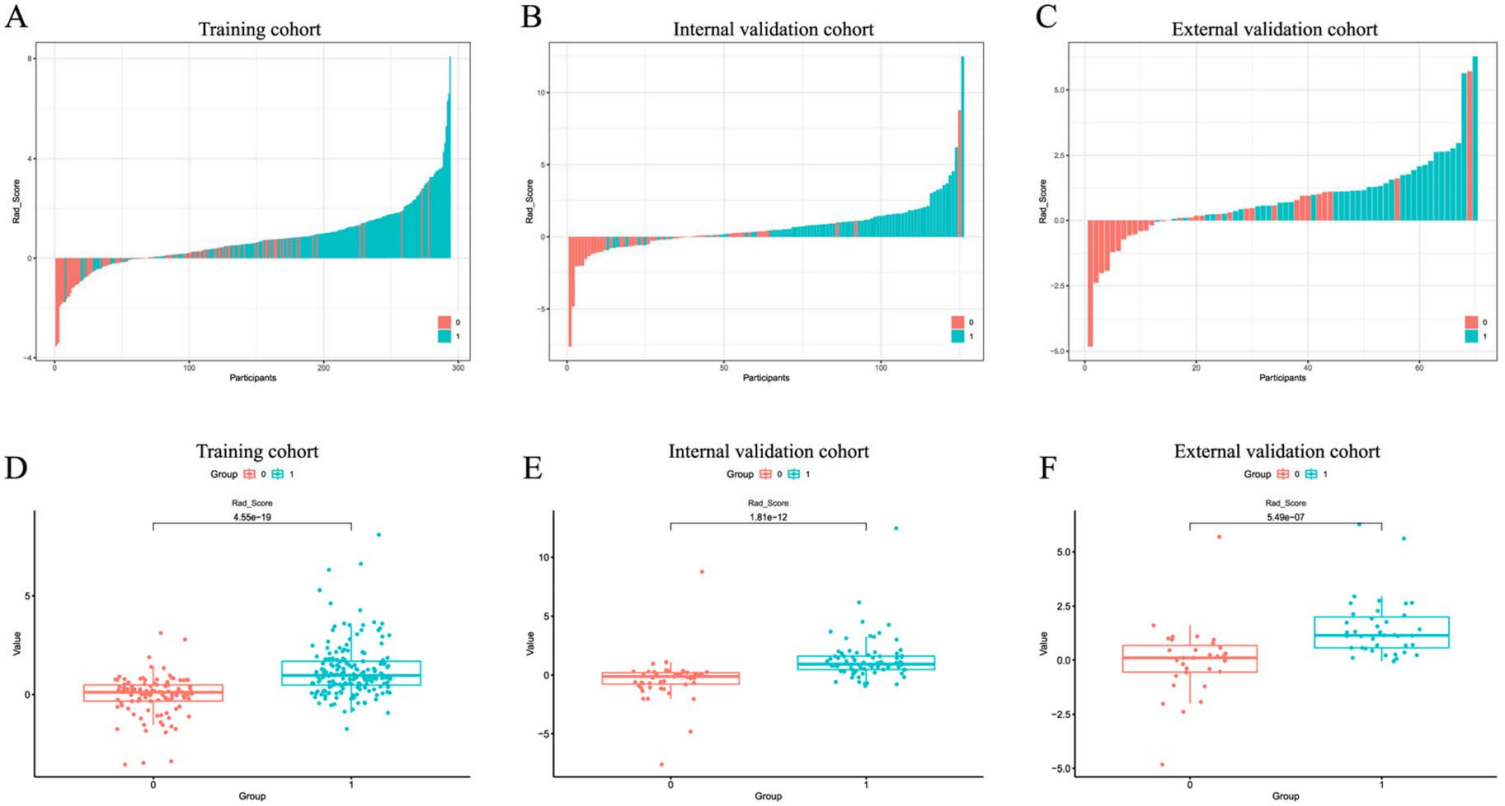
Evaluation of the Radiomic Score for extrathyroidal extension across cohorts. Distribution and comparative analysis of the Radiomic Score (Rad-score) across cohorts. (a–c) The distribution of the Rad-score in the training, internal validation, and external validation cohorts, respectively. (d–f) Box plots comparing the Rad-scores between tumors with and without extrathyroidal extension (ETE) in the training, internal validation, and external validation cohorts, respectively. The Rad-score was significantly higher in the ETE group compared to the non-ETE group across all three cohorts (all p-values < 0.05).

**Figure 4.**
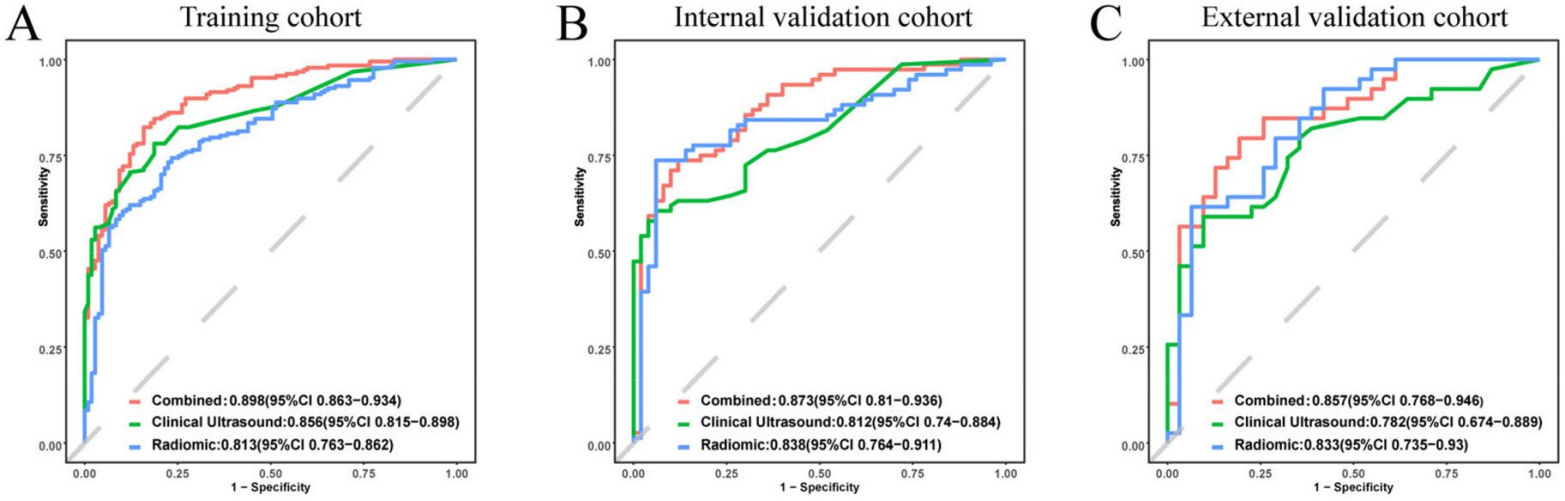
Assessment of model performance for predicting extrathyroidal extension (ETE). (a–c) Receiver operating characteristic (ROC) curves of the radiomic model, clinical model, and combined model in the training, internal validation, and external validation cohorts, respectively.

The distribution of the radiomics score (Rad-score) and extrathyroidal extension (ETE) status across the three cohorts is illustrated in Figure 3. The Mann-Whitney U test revealed a statistically significant difference in Rad-scores between the ETE and non-ETE groups (*p* < 0.001). Furthermore, univariate and multivariate regression analyses identified the Rad-score as an independent factor associated with ETE. Multivariate analysis confirmed C-TIRADS 4c, age ≥55 years, lateral lymph node metastasis, nodule contact with the thyroid capsule, tumor-tracheal angle, contact with the tracheoesophageal groove as independent predictors of ETE, which were subsequently incorporated into the clinical model. Finally, the combined model was developed by integrating the Rad-score with these independent clinical predictors (Table 2). Figure 4 displays the performance of the radiomics, clinical, and combined models. The constructed radiomics model, comprising seven selected features, demonstrated good discriminatory performance, with areas under the curve (AUC) of 0.813 (95% CI: 0.763–0.862), 0.838 (95% CI: 0.764–0.911), and 0.833 (95% CI: 0.735–0.930) in the training, internal validation, and external validation cohorts, respectively. The seven features were: original_firstorder_Minimum, original_glcm_ClusterShade, original_glrlm_LongRunHighGrayLevelEmphasis, original_glszm_GrayLevelNonUniformity, original_glszm_SizeZoneNonUniformityNormalized, original_glszm_ZoneVariance, and wavelet.LH_glszm_LowGrayLevelZoneEmphasis. These features represent first-order statistics, texture characteristics (GLCM, GLRLM, GLSZM), and wavelet-transformed features, capturing intensity distribution, spatial heterogeneity, and frequency-domain information of the tumor on ultrasound images.

According to the DeLong test, the differences in AUC between the combined model and the clinical model were statistically significant in the training and internal validation cohorts (*p* = 0.001 and *p* = 0.03, respectively). In the external validation cohort, the combined model achieved an AUC of 0.857 (95% CI: 0.768–0.946) compared to 0.782 (95% CI: 0.674–0.889) for the clinical model, although this difference was not statistically significant (*p* = 0.11). The DeLong test also indicated a statistically significant AUC difference between the combined model and the radiomics model in the training cohort [AUC = 0.898 (95% CI: 0.863–0.934) vs. 0.813 (95% CI: 0.763–0.862); *p* < 0.001]. Nevertheless, no statistically significant differences were detected in the internal validation cohort [AUC = 0.873 (95% CI: 0.810–0.936) vs. 0.838 (95% CI: 0.764–0.911); *p* = 0.28] or the external validation cohort [AUC = 0.857 (95% CI: 0.768–0.946) vs. 0.833 (95% CI: 0.735–0.930); *p* = 0.56].

### Nomogram construction and performance

**Figure 5.**
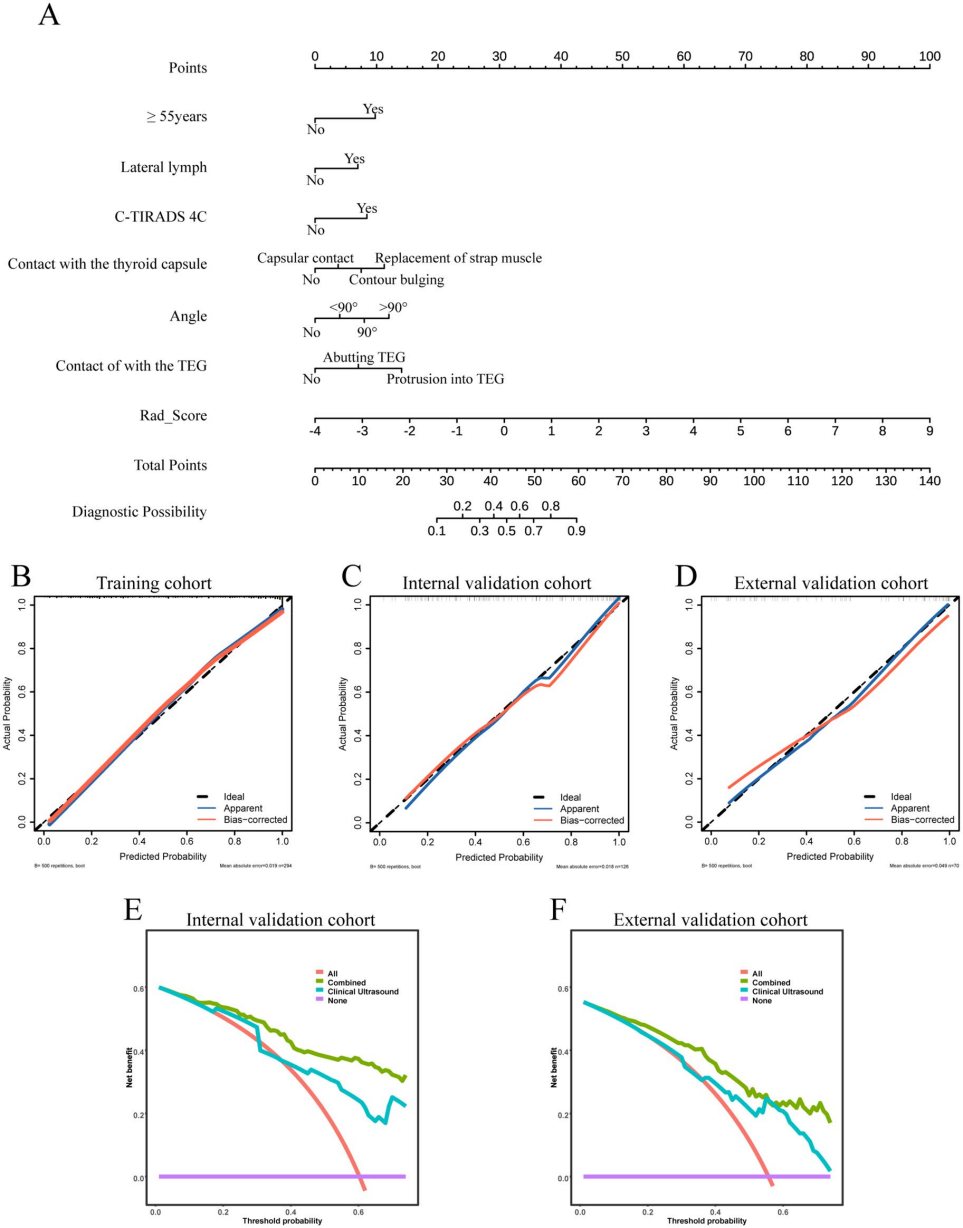
Development and performance of radiomic nomogram. (a) The radiomic nomogram. (b–d) Calibration curves assessing the agreement between predicted probability and actual occurrence of ETE across the training, internal, and external validation cohorts. (e, f) Decision curve analysis evaluating the net benefit of the combined model (green), clinical-US model (blue), versus the ‘all’ (red) or ‘none’ (purple) strategies in the internal and external test sets.

The nomogram, which integrates radiomics features and common clinical factors into a visual tool, serves as a valuable instrument for facilitating clinical consultation and patient education. The combined model was transformed into a nomogram, where the total points derived from the nomogram correspond to the predicted risk of ETE occurrence (Figure 5(a)). The calibration curves demonstrated good agreement between the predictions of the combined model and the actual observed ETE status across all three cohorts **(**Figure 5(b–d)). The Brier score provided key quantitative evidence for this, with values of 0.123, 0.134, and 0.169 for the training, internal validation, and external validation cohorts, respectively—all consistently low. This quantitatively demonstrates the excellent calibration and overall accuracy of the model’s predicted probabilities. Decision curve analysis further indicated that the combined model yielded higher overall net benefit across most threshold probabilities in both the internal and external validation sets **(**Figure 5(e, f)). A summary of the predictive performance for all three models is provided in Table 3.

**Table 3.** Comparison of diagnostic performance among radiomics model, clinical model, and combined model in training cohort, internal validation cohort, and external validation cohort.

Model	AUC (95%CI)	Accuracy (95%CI)(%)	Sensitivity (%)	Specificity (%)	PPV (%)	NPV (%)
Radiomic model						
Training cohort	0.813(0.763–0.862)	75.2(69.8–80.0)	74.3	76.6	84.8	63.1
Internal validation cohort	0.838(0.764–0.919)	78.6(70.4–85.4)	68.4	94.0	94.6	66.2
External validation cohort	0.833(0.735–0.930)	75.7(64.0–85.2)	79.5	71.0	77.5	73.3
Clinical model						
Training cohort	0.856(0.815–0.898)	79.3(74.2–83.7)	78.1	81.3	88.0	68.0
Internal validation cohort	0.812(0.740–0.884)	71.4(62.7–79.1)	72.4	70.0	78.6	62.5
External validation cohort	0.782(0.674–0.889)	68.6(56.4–79.2)	84.6	48.4	67.4	71.4
Combined model						
Training cohort	0.898(0.863–0.934)	83.0(78.2–87.1)	82.4	84.1	90.1	73.1
Internal validation cohort	0.873(0.810–0.937)	78.6(70.4–85.4)	73.7	86.0	88.9	68.3
External validation cohort	0.857(0.768–0.946)	74.3(62.4–84.0)	84.6	61.3	73.3	76.0

Abbreviations: AUC (Area Under the Curve); CI (Confidence Interval); PPV (Positive Predictive Value); NPV (Negative Predictive Value).

### Subgroup analysis

The results of the subgroup analysis are summarized in Table 4. The combined model demonstrated discriminative ability for ETE across all subgroups stratified by various clinical characteristics.

**Table 4.** Performance metrics of the clinicopathological-ultrasonographic model for predicting ETE of thyroid cancer in different patient subgroups, along with intra-subgroup comparisons using the Hanley-McNeil Z-test.

	AUC (95% CI)	Accuracy	Sensitivity	Specificity	*p*
Cohort 1					
Gross ETE	0.966(0.942–0.990)	0.874	0.953	0.842	< 0.001
Minimal ETE	0.866(0.829–0.904)	0.792	0.749	0.847	
Cohort 2					
Microcarcinomas	0.834(0.785–0.882)	0.768	0.658	0.863	0.565
non-microcarcinomas	0.870(0.755–0.986)	0.894	0.916	0.722	
Cohort 3					
Hashimoto’s thyroiditis	0.907(0.866–0.948)	0.814	0.797	0.845	0.270
Non-Hashimoto’s thyroiditis	0.879(0.833–0.924)	0.819	0.800	0.849	
Cohort 4					
Euthyroid	0.891(0.848–0.934)	0.797	0.788	0.810	0.871
Thyroid dysfunction	0.896(0.852–0.941)	0.835	0.807	0.890	

Abbreviations: AUC (area under the receiver operating characteristic curve); ETE (extrathyroidal extension).

*p*-values correspond to the comparison of the AUCs between subgroups, calculated using the Hanley–McNeil Z-test.

In the subgroup stratified by the extent of invasion, the combined model achieved an area under the curve (AUC) of 0.966 (95% CI: 0.942–0.990) in the gross ETE subgroup and 0.866 (95% CI: 0.829–0.904) in the minimal ETE subgroup, with a statistically significant difference in AUC between the two subgroups (*p* < 0.001). In the tumor size subgroup, the model yielded an AUC of 0.834 (95% CI: 0.785–0.882) in the microcarcinoma subgroup and 0.870 (95% CI: 0.755–0.986) in the non-microcarcinoma subgroup, with no significant intergroup difference (*p* = 0.565).

Furthermore, model performance was comparable between subgroups with and without Hashimoto’s thyroiditis (AUC: 0.907, 95% CI: 0.866–0.948 vs. AUC: 0.879, 95% CI: 0.833–0.924; *p* = 0.270). Similarly, no statistically significant difference in performance was observed between subgroups with normal thyroid function (AUC: 0.891, 95% CI: 0.848–0.934) and those with abnormal thyroid function (AUC: 0.896, 95% CI: 0.852–0.941; *p* = 0.871).

Secondary performance metrics, including accuracy, sensitivity, and specificity for each subgroup, are detailed in Table 4.

## Discussion

This study systematically developed and validated a combined model for the preoperative prediction of extrathyroidal extension (ETE) in PTC, based on a cohort of 420 patients for training and internal validation, alongside an independent external test cohort of 70 patients. The model integrates seven key radiomics features, C-TIRADS 4c, age ≥55 years, lateral lymph node metastasis, and anatomical characteristics such as the angle between tumor and trachea. Validation results demonstrated that the combined model achieved an AUC of 0.898 (95% CI: 0.863–0.934) in the training cohort, significantly outperforming both the clinical model (*p* = 0.001) and the radiomics model (*p* < 0.001). It maintained strong performance in the internal validation cohort with an AUC of 0.873 (95% CI: 0.810–0.936), remaining significantly superior to the clinical model (*p* = 0.03), and exhibited robust generalizability in the external independent validation (AUC = 0.857). Furthermore, subgroup analysis confirmed the model’s consistent performance across key subgroups. It is noteworthy that the model demonstrated favorable AUCs for both minimal ETE (AUC 0.866) and gross ETE (AUC 0.966) within the subgroup categorized by the extent of invasion, although the intergroup comparison showed a statistically significant difference (*p* < 0.001). In contrast, no significant differences in model performance were observed among subgroups stratified by tumor size or Hashimoto’s thyroiditis status (all *p* > 0.05). These findings indicate that the combined model established in this study provides an objective, robust, and multidimensional predictive tool for preoperative ETE risk assessment in PTC patients.

According to the National Comprehensive Cancer Network (NCCN) Guidelines, gETE represents a clear indication for total thyroidectomy. gETE is clinically significant due to its substantial impact on patient prognosis. Evidence indicates that patients with gETE experience higher rates of tumor recurrence, shorter overall survival, and an incidence of adverse outcomes exceeding 50%. Similarly, the risks of lymph node metastasis and distant metastasis are significantly elevated compared to those without ETE. In contrast, the clinical significance of mETE remains controversial. Although the 8th edition of the AJCC Cancer Staging Manual has removed mETE from the definition of pT3 disease, the American Thyroid Association guidelines still classify it as an intermediate-risk category for recurrence [19]. Moreover, multiple studies have confirmed that mETE is an independent predictor of persistent/recurrent disease in patients with PTC and is associated with shortened disease-free survival [20,21]. Given this established association between mETE, gETE, and their impact on clinical prognosis, our study categorizes both as ETE for subsequent analysis.

It is imperative to emphasize that accurate assessment of ETE is crucial for precise thyroid cancer staging. The ETE status serves not only as a critical determinant for evaluating the appropriateness of the extent of surgical resection and the potential need for active surveillance strategies but also provides valuable reference for surgeons to formulate individualized preoperative plans. Consequently, this approach helps prevent patients from undergoing unnecessary extensive surgery, thereby ensuring therapeutic efficacy while mitigating risks associated with overtreatment.

Our findings reveal that both age ≥55 years and lateral lymph node metastasis (LLNM) are significantly associated with ETE in patients with PTC. Patients aged ≥55 years demonstrated an odds ratio (OR) of 2.6 for ETE, while those with LLNM showed an OR of 3.8, suggesting both parameters may serve as potential risk factors for ETE and provide clinical reference for identifying high-risk populations. Regarding the association between age and ETE, our observations align with existing literature. For instance, Qi et al. [22] previously established a correlation between age and ETE, while Kuo et al. [23] further identified age (OR = 1.025) as an independent risk factor through multivariate analysis. By establishing 55 years as the cutoff value, our study more precisely captures the significantly elevated ETE risk (OR = 2.6) in older patients (≥55 years). This discrepancy likely stems from different age stratification criteria, underscoring the clinical significance of ETE risk assessment in specific age cohorts.

LLNM demonstrated a significant correlation with ETE. A meta-analysis encompassing 15 studies and 8,369 clinically node-negative PTC patients revealed a significant association between ETE and LLNM (OR = 2.52) [24]. Similarly, Song et al. [25] confirmed ETE as an independent risk factor for lymph node metastasis (OR = 1.852). Our study, from a reverse perspective, found that patients with LLNM had an OR of 3.8 for ETE occurrence. These findings collectively form a bidirectional association between ETE and LLNM, suggesting that the presence of LLNM in PTC patients should raise clinical suspicion for ETE. This warrants thorough preoperative imaging evaluation or intraoperative exploration to delineate the extent of invasion, thereby optimizing surgical decision-making and reducing recurrence risk.

Furthermore, this study found that some clinically significant variables identified in the univariate analysis (such as multifocality) were not retained in the final multivariate model. This may be attributed to collinearity among variables, or because their predictive information was already encompassed by other more representative variables in the model (such as lateral cervical lymph node metastasis and contact of the nodule with the thyroid capsule). Multivariate modeling aims to identify independent predictors; therefore, it preferentially retains variables with the highest contribution and strongest clinical interpretability.

Previous studies have established that specific US features suggesting tumor invasion of the strap muscles, trachea, or RLN hold reference value for the preoperative diagnosis of ETE [9,26,27]. Our results further substantiate this conclusion: multivariate logistic regression analysis identified contact of the nodule with the thyroid capsule (OR = 2.5) and contact of the nodule with the TEG structures (OR = 2.2) as independent predictors for ETE. These findings are largely consistent with previously reported data, reaffirming the clinical significance of these US characteristics in preoperative ETE assessment. Building upon this foundation, our study expands the dimensions of US evaluation. We integrated anatomical features related to the tumor margin on US images – such as angle between tumor and trachea and contact of the nodule with the TEG – with the Chinese Thyroid Imaging Reporting and Data System (C-TIRADS) classifications and relevant clinicopathological factors. This integration culminated in a novel assessment strategy that synthesizes anatomical characteristics, imaging classifications, and biological behavior, providing a new perspective for a more comprehensive and precise preoperative prediction of ETE in clinical practice.

Understanding cancer-related biological behavior and molecular biology has traditionally relied on invasive biopsy procedures. In contrast, radiomics emerges as a rapid, cost-effective, and non-invasive imaging biomarker [28–30], potentially offering a promising alternative to biopsy for tumor evaluation. Furthermore, radiomics can overcome the subjectivity inherent in conventional image interpretation by providing quantitative imaging features [31–33], thereby opening new frontiers in oncologic imaging. It possesses considerable potential for extracting valuable medical insights and improving the precision of clinical differential diagnosis. A previous investigation has demonstrated the utility of ultrasound-derived radiomic features in assessing ETE [34]. In our investigation, we initially extracted 849 potential radiomic features from ultrasound images. Following evaluation by intraclass correlation coefficient (ICC) and feature screening, we employed the Least Absolute Shrinkage and Selection Operator (LASSO) regression model for further dimensionality reduction, ultimately identifying 7 core radiomic features as predictors for constructing the ETE prediction model. The selected radiomic features were categorized into three types (first-order, texture, and wavelet features), all demonstrating significant differences between the non-ETE and ETE groups. These features essentially capture information derived from pixel intensity and texture patterns within ultrasound images. First-order features primarily characterize the internal textural properties of lesions. Texture features, including those derived from the gray-level co-occurrence matrix, gray-level run-length matrix, and gray-level size zone matrix, delineate the spatial relationships between pixels and their adjacent neighbors. Wavelet features mainly reflect time-frequency domain characteristics within the lesion[35]. Among the selected ultrasound radiomic features, the wavelet feature ‘wavelet.LL_glszm_LowGrayLevelZoneEmphasis’ and the texture features ‘original_glszm_GrayLevelNonUniformity’ and ‘original_glszm_SizeZoneNonUniformityNormalized’ demonstrated high significance and robustness in predicting ETE. They characterize the time-frequency domain properties and heterogeneity of spatial texture distribution within the lesion area on ultrasound images, respectively. To some extent, ultrasound radiomics itself constitutes a quantitative methodology. Quantitative ultrasound assessment has been applied in various aspects of ETE diagnosis, risk stratification, and treatment planning. Jiang et al. [18] reported an evaluation model based on contrast-enhanced ultrasound (CEUS) characteristics, which incorporated key features from imaging and clinical data, utilizing radiomic analysis as a predictive tool for ETE in PTC. They found their integrated model outperformed models constructed using single quantitative parameters alone. Our study similarly established a model combining clinical, conventional ultrasound, and radiomic features. Notably, our combined model achieved an AUC of 0.898 in the test cohort, significantly outperforming models based solely on individual US or clinical features. These results confirm the superiority of multi-modal models in diagnosing ETE. While clinicians often tailor treatment strategies for PTC patients based on clinical factors like age, our multi-modal radiomic-clinical model (AUC = 0.873 in the test cohort) demonstrated superior performance compared to the prediction model relying solely on clinical features (AUC = 0.782). Furthermore, in the external validation set, our combined model maintained robust performance with an AUC of 0.857, outperforming single-modality models, although the differences were not statistically significant.

To further validate the applicability of our model, we considered that different disease backgrounds and tumor characteristics might significantly influence the manifestations and diagnostic challenges of ETE [36,37]. We therefore investigated the performance of the combined model across four distinct patient subgroups. Subgroup analysis demonstrated that the combined model exhibited extensive and robust generalizability in predicting ETE. Across subgroups with varying degrees of invasion, the model maintained high predictive capability for both gross ETE (AUC = 0.966) and minimal ETE (AUC = 0.866), although a statistically significant difference in AUC was observed between these groups (*p* < 0.001). In tumor size-based subgroups, the model demonstrated comparable predictive efficacy for both microcarcinomas (AUC = 0.834) and non-microcarcinomas (AUC = 0.870) (*p* = 0.565). Furthermore, the model consistently maintained stable classification performance regardless of Hashimoto’s thyroiditis status (AUC: 0.907 vs. 0.879, *p* = 0.270) or thyroid function status (AUC: 0.891 vs. 0.896, *p* = 0.871). Metrics including accuracy, sensitivity, and specificity remained consistent across all subgroups. In summary, the combined model demonstrated consistent and reliable predictive performance across multiple clinical subgroups stratified by invasion extent, tumor size, Hashimoto’s thyroiditis status, and thyroid function, indicating its broad potential for clinical application.

However, this study has several limitations. Its retrospective multicenter design introduces the possibility of sample selection bias. Future studies should incorporate larger, prospectively collected multicenter datasets to enhance sample representativeness. Secondly, the assessment of ultrasound features utilized by the model—such as contact of the nodule with the thyroid capsule and the angle between tumor and trachea—remains partially dependent on operator experience and judgment, introducing a degree of subjectivity. The inter-observer and intra-observer consistency of these evaluations requires further standardization and validation in prospective settings. Moving forward, we plan to conduct large-scale, prospective multicenter studies to validate and refine this model, while also exploring its integration with artificial intelligence-assisted diagnostic systems to improve both objectivity and clinical utility.

## Conclusion

In summary, the ultrasound radiomics-based combined model demonstrates excellent diagnostic performance for predicting extrathyroidal extension in thyroid cancer. It maintains robust predictive capability not only in the overall cohort but also across key clinical subgroups stratified by invasion extent, tumor size, concomitant Hashimoto’s thyroiditis, and thyroid functional status. With the advancing integration of artificial intelligence in ultrasound diagnostics, this quantifiable and visually accessible nomogram model shows significant potential for clinical translation. Future research should focus on its validation in prospective cohorts and integration into clinical workflows, thereby supporting automated and precise ultrasound assessment of ETE in thyroid cancer patients.

## Data Availability

The data that support the findings of this study are available from the corresponding author (P.X., xupan_1989@126.com) upon reasonable request and with the approval of the ethics committee at the First Affiliated Hospital of Nanchang University.
